# Retrospective Cohort Study on the Incidence and Management of Hemiplegic Shoulder Pain in Stroke Inpatients

**DOI:** 10.7759/cureus.76030

**Published:** 2024-12-19

**Authors:** Igor Santos Neto, Miguel Guimaraes, Tiago Ribeiro, Ana Gonçalves, Ines Natario, Marta Torres

**Affiliations:** 1 Physical Medicine and Rehabilitation, Centro de Reabilitação do Norte - Centro Hospitalar de Vila Nova de Gaia, Vila Nova de Gaia, PRT; 2 Physical Medicine and Rehabilitation, Centro de Reabilitação do Norte, Vila Nova de Gaia, PRT

**Keywords:** cerebrovascular accident, corticosteroid injections, inpatient rehabilitation, painful hemiplegic shoulder, pain management, post-stroke complications, pulsed radiofrequency, rehabilitation outcomes, shoulder pain, stroke rehabilitation

## Abstract

Background: Painful hemiplegic shoulder (PHS) is a prevalent and challenging complication following a stroke and can significantly impair a patient's engagement in rehabilitation, leading to poorer functional outcomes and extended hospital stays. This retrospective cohort study aims to investigate the incidence, etiology, and management of PHS in stroke inpatients, focusing on the effectiveness of various therapeutic interventions.

Methods: We conducted a retrospective analysis of subacute stroke inpatients who developed PHS during rehabilitation at a single center. Medical records were reviewed to assess the incidence of PHS, underlying causes, and treatment modalities. Primary outcome measures included the prevalence of PHS, the distribution of identified etiologies, and therapeutic outcomes associated with different management strategies.

Results: Our findings revealed a significant prevalence of PHS among stroke inpatients, consistent with existing literature. The multifactorial etiology included spasticity, adhesive capsulitis, glenohumeral subluxation, central post-stroke pain, and complex regional pain syndrome, with advanced age, low functional scores, motor and sensory impairments, and comorbidities such as diabetes mellitus identified as key risk factors. Management strategies ranged from conservative approaches, such as physical modalities and slings, to advanced interventions, including intra-articular corticosteroid injections, botulinum toxin type A applications, nerve blocks, and radiofrequency neuromodulation. Corticosteroid injections and electrical stimulation were particularly effective in alleviating pain and improving functional outcomes. Notably, pulsed radiofrequency modulation targeting the suprascapular and axillary nerves showed superior efficacy in enhancing the passive range of motion compared to conventional nerve blocks, although the effectiveness of botulinum toxin type A was inconsistent.

Conclusions: This study emphasizes the multifaceted nature of PHS in stroke inpatients, underlining the importance of individualized and comprehensive treatment strategies. While several therapeutic interventions, particularly corticosteroid injections and pulsed radiofrequency, demonstrated effectiveness, the variability in treatment outcomes highlights the need for further investigation. Future research should focus on larger patient cohorts with extended follow-up periods to better elucidate the progression of PHS and refine management approaches. Despite limitations, including the retrospective study design and a short follow-up period, these findings provide valuable insights into the prevalence, progression, and treatment of PHS in stroke rehabilitation.

## Introduction

Stroke remains a leading cause of mortality and disability, imposing significant socioeconomic and healthcare burdens on developed countries. Continuous advancements in treatment options have underscored the importance of early rehabilitation programs in enhancing functional independence and improving patient outcomes. However, the rehabilitation process is often hindered by complications, necessitating a comprehensive understanding of strategies to address these challenges [1].

One such complication is painful hemiplegic shoulder (PHS), a condition characterized by shoulder pain following a cerebrovascular accident. Key risk factors for PHS include reduced motor function, type 2 diabetes mellitus (DM2), and a history of prior shoulder pain [2]. Incidence rates of PHS vary widely, from 9% to 73% in earlier reports [3] to more recent findings of 24-64% in inpatient rehabilitation settings [4]. PHS onset varies from two weeks to three months post-stroke, reflecting the heterogeneity of this condition [5]. This condition significantly impairs patients' participation in rehabilitation, resulting in lower Barthel scores at discharge, poorer functional recovery, and extended hospital stays [6].

The multifactorial etiology of PHS includes both musculoskeletal and neurological changes. Common contributors are spasticity, adhesive capsulitis, glenohumeral subluxation, central post-stroke pain, and complex regional pain syndrome [7]. Risk factors such as advanced age, low functional scores, dependence on transfers, neglect, sensory changes, and comorbidities like diabetes mellitus or depression further complicate the clinical picture [8].

Managing PHS is a significant clinical challenge, but effective treatment can enhance patients' participation in rehabilitation, leading to better functional outcomes. Treatment modalities range from conservative approaches, such as physical modalities and slings, to minimally invasive techniques, including intra-articular corticosteroid injections, botulinum toxin injections, nerve blocks, and radiofrequency neuromodulation [9].

Study aim

Given the diverse etiological factors and management strategies for PHS, this retrospective study aimed to determine the prevalence of each identified cause and evaluate the range of treatment options implemented in an inpatient rehabilitation setting.

## Materials and methods

This study utilised a retrospective cohort design to examine the incidence, aetiology, and management of PHS in stroke inpatients admitted to the North Rehabilitation Centre between July 2021 and July 2023. The research protocol adhered to institutional ethics guidelines, with patient confidentiality maintained throughout.

Patients included in the study were adults aged 18 years or older who were diagnosed with PHS during their inpatient rehabilitation stay. All participants were post-stroke individuals confirmed via imaging (CT or MRI) with hemiparesis or hemiplegia and demonstrated the cognitive ability to respond to health-related questions or had caregivers capable of providing reliable patient histories. Additionally, patients provided informed consent for the use of their medical data in research.

Exclusion criteria were patients with pre-existing shoulder pathologies unrelated to their stroke, such as severe osteoarthritis or shoulder dislocations not associated with the cerebrovascular event. Individuals with comorbid conditions significantly confounding the diagnosis of PHS, including active systemic inflammatory diseases (e.g., rheumatoid arthritis) or chronic widespread pain syndromes, were also excluded. Further exclusions applied to those unable to participate in rehabilitation due to severe cognitive or communication impairments unrelated to stroke (e.g., severe dementia, global aphasia), patients treated exclusively on an outpatient basis without adequate inpatient rehabilitation data, and individuals with incomplete or missing clinical records that prevented reliable assessment of PHS diagnosis, aetiology, or treatment outcomes.

Data collection

Patient data were extracted from the centre’s electronic medical records, including the Stroke Rehab Unit Database, therapeutic intervention sheets, and radiological reports. Clinical records were reviewed to identify the incidence of PHS diagnoses during inpatient rehabilitation document underlying causes, including musculoskeletal and neurological aetiologies, and detail the therapeutic interventions employed and their corresponding outcomes. Collected variables included demographic data (age, sex, BMI), comorbidities (e.g., diabetes mellitus, hypertension, dyslipidemia), stroke-specific data (type of stroke (ischaemic or haemorrhagic)), location of brain lesion, and functional impairment at admission, pHS-specific factors (timing of onset, associated aetiologies; e.g., spasticity, adhesive capsulitis, glenohumeral subluxation), and severity of pain (using validated pain scales). The rehabilitation outcomes were measured with Functional Independence Measure (FIM) scores at admission and discharge, length of hospital stay, and dependency in activities of daily living (ADLs).

Data sources and confidentiality

The study period was selected based on the availability of complete medical records and consistency in the rehabilitation protocols during this timeframe. Data were extracted from the Stroke Rehab Unit Database, clinical records, therapeutic documentation, and radiological reports within the study period. Diagnoses contributing to PHS included adhesive capsulitis, rotator cuff tendinopathy, glenohumeral subluxation, and other documented aetiologies. To ensure confidentiality, all data were anonymized using patient record numbers, and information was randomly ordered by the database system to prevent identification.

Statistical analysis

Statistical analyses were conducted using SPSS software (IBM Corp., Armonk, NY). The incidence of PHS was calculated as a proportion of the total cohort. Univariate and multivariate logistic regression analyses were performed to identify risk factors associated with PHS development. Continuous variables (e.g., FIM scores) were compared using the Mann-Whitney U test, while categorical variables (e.g., presence of spasticity) were compared using chi-square tests. P-values <0.05 were considered statistically significant.

Outcome measures

The primary outcome measures were the prevalence of PHS among stroke inpatients, distribution of identified aetiologies, and functional outcomes associated with different therapeutic interventions. The secondary outcome measures included associations between demographic and clinical variables with PHS incidence and the effectiveness of specific therapeutic modalities in reducing pain and improving functional outcomes.

Rationale for exclusion criteria

The exclusion criteria were designed to enhance the validity of findings by minimising confounding factors. Pre-existing or unrelated shoulder pathologies could obscure the contribution of stroke-related factors to PHS development. Similarly, excluding patients with incomplete data ensured the reliability of the dataset, while excluding outpatient-only cases focused the study on the inpatient rehabilitation context. This comprehensive methodological framework aimed to provide robust insights into the complex interplay between PHS incidence, aetiology, and management, offering a foundation for future prospective studies.

## Results

In this study, post-stroke shoulder pain was identified in 125 patients, representing 31.2% of the total cohort of 401 stroke inpatients. The causes of PHS were diverse and multifactorial. The most common diagnoses associated with this condition included multifactorial etiologies (n=40, 10%), glenohumeral subluxation (n=25, 6.2%), rotator cuff tendinopathies (n=20, 5%), spasticity (n=18, 4.4%), adhesive capsulitis (n=13, 3.2%), complex regional pain syndrome (n=6, 1.5%), and central post-stroke pain (n=2, 0.5%) (Table 1).

**Table 1:** Prevalence and Etiological Breakdown of PHS in Stroke Patients n=number of patients; PHS: painful hemiplegic shoulder

Variables	N (%)
Total number of stroke patients	401
Patients with painful hemiplegic shoulder	125 (31.2%)
Multifactorial causes	40 (10%)
Glenohumeral subluxation	25 (6.2%)
Rotator cuff tendinopathies	20 (5%)
Spasticity	18 (4.4%)
Adhesive capsulitis	13 (3.2%)
Complex regional pain syndrome	6 (1.5%)
Central pain	3 (0.5%)

PHS is a complex condition that requires an individualized and multifaceted approach due to the diversity in its etiological factors and therapeutic interventions. The management of PHS often involves conventional physiatric treatments, which encompass physical modalities and therapeutic exercises. These were the most frequently utilized strategies, applied in 111 patients (27.7%). Additional therapeutic interventions included intra-articular corticosteroid and anaesthetic injections (n=35, 8.7%), ultrasound-guided botulinum toxin injections (n=20, 5%), suprascapular nerve blocks (n=27, 6.7%), and radiofrequency neuromodulation treatments (n=11, 2.7%) as described in Table 2. These varied approaches highlight the need for an adaptable therapeutic plan that can address the unique needs and conditions of each patient. The data in Table 2 further details these interventions, emphasizing the breadth of available treatment options for this challenging condition.

**Table 2:** Therapeutic Interventions for PHS in Stroke Rehabilitation n=number of patients; PHS: painful hemiplegic shoulder

Therapeutic Interventions	N (%)
Conventional physiatric treatment	111 (27.7%)
Intra-articular corticosteroid and anesthetic injection	35 (8.7%)
Ultrasound-guided botulinum toxin injection	20 (5%)
Suprascapular nerve block	27 (6.7%)
Radiofrequency	11 (2.7%)

The analysis of patient characteristics revealed significant associations with the incidence of PHS. Hypoesthesia was found to be significantly associated with a higher incidence of PHS (p = 0.013), indicating that sensory deficits may play a critical role in its development. Similarly, patients with unilateral motor alterations demonstrated a higher incidence of shoulder pain compared to those with bilateral motor alterations (p = 0.003), suggesting that the nature and extent of motor dysfunction significantly influence the risk of developing PHS. Interestingly, patients with normal body weight showed a higher incidence of PHS than obese patients (p = 0.017), raising questions about the role of physical activity and body composition.

Functional outcomes were markedly poorer in patients with PHS. These patients demonstrated significantly lower motor and total scores on the Functional Independence Measure (FIM) (p < 0.001), reflecting the substantial impact of PHS on rehabilitation progress and overall functionality. Furthermore, PHS was associated with significantly higher dependency levels in various transfer activities, including transfers from bed (p = 0.035), shower (p = 0.008), and bathroom (p = 0.023). These dependencies underscore the far-reaching effects of PHS on daily living activities and the challenges it poses to achieving independence during rehabilitation, as described in Table 3. Analysis of comorbidities, including dyslipidemia, sleep apnea, hypertension, and diabetes mellitus, revealed no statistically significant associations with the incidence of PHS or functional outcomes. Nonetheless, a trend was observed among older patients (≥75 years), suggesting a potential age-related risk factor for PHS development that warrants further investigation (p = 0.070).

**Table 3:** Associations Between Patient Characteristics and the Incidence of PHS FIM scores: Functional Independence Measure scores; PHS: painful hemiplegic shoulder A p-value of < 0.05 was considered statistically significant. ^*^Chi-square test; ^&^Mann-Whitney U test

Patient Characteristics	P-value
Patients with hypoesthesia had a higher incidence of PHS	0.013^*^
Motor alterations (unilateral or bilateral): patients with unilateral alterations showed a higher incidence of shoulder pain compared to those with bilateral alterations.	0.003^*^
Patients with normal body weight had a higher incidence of PHS compared to obese patients.	0.017^*^
Patients with PHS had lower motor and total FIM scores in comparison.	<0.001^&^
Patients with PHS exhibited significantly higher levels of dependency, particularly in transfers such as:	
Bed	0.035^&^
Shower	0.008^&^
Bathroom	0.023^&^

In summary, these findings highlight the complex interplay of etiological factors, therapeutic approaches, and functional outcomes in managing PHS. The significant diversity in causes and treatments emphasizes the need for a multifaceted and individualized approach, tailoring interventions to the specific characteristics and conditions of each patient.

## Discussion

This study investigated the incidence and management of PHS in a cohort of subacute post-stroke patients admitted to a rehabilitation centre, highlighting this condition as a prevalent post-stroke complication.

The reported incidence of PHS varies widely across studies due to differences in the definitions of shoulder pain and diagnostic criteria employed [10]. Our findings indicate that between 29% [11] and 44% [12] of stroke patients in inpatient rehabilitation developed PHS, aligning with existing literature reporting incidences as high as 60% in four-month follow-ups [13]. This high prevalence underscores the urgent need for robust prevention and management strategies tailored to this population. Variability in reported incidence rates is partly explained by differing definitions and diagnostic criteria for hemiplegic shoulder pain across studies.

Our study reveals that approximately 31% of stroke patients admitted to inpatient rehabilitation developed PHS during the follow-up period. This finding is consistent with previous literature reporting similar rates [4]. Given the high prevalence, there is a pressing need for prevention and management strategies to address the complexity of PHS and the interplay of musculoskeletal and neurological factors in its development [14]. The diversity in treatment modalities reflects this complexity and emphasizes the necessity for individualized care, accounting for distinct etiologies and varying patient responses to therapy, as documented in previous studies [15,16].

In this study, significant associations were observed between PHS and various patient characteristics. Notably, patients with hypoesthesia demonstrated a higher incidence of PHS (p = 0.013), indicating that sensory deficits may increase vulnerability to shoulder pain. This finding aligns with previous systematic reviews [4,17], supporting the hypothesis that PHS has neuropathic components in its mechanism [18]. Furthermore, patients with unilateral motor changes exhibited a greater incidence of PHS compared to those with bilateral impairments (p = 0.003), suggesting that the degree and nature of motor dysfunction influence pain outcomes, consistent with earlier studies [2,4].

Interestingly, patients with normal body weight had a higher incidence of PHS compared to obese patients (p = 0.017). This finding warrants further investigation into the role of body composition and weight in PHS development, potentially reflecting differing levels of physical activity or functional mobility. PHS significantly impacted functional outcomes. Patients with this condition exhibited lower scores on the FIM scale, both for motor and total scores (p < 0.001), findings that mirror those reported in other studies [13,19]. Additionally, patients with PHS demonstrated increased dependency in transfers, such as moving from bed to shower or bathroom (p = 0.035). These results align with existing evidence highlighting the detrimental effects of PHS on quality of life and rehabilitation outcomes [11].

PHS is associated with poorer rehabilitation outcomes and diminished quality of life, as demonstrated in previous studies [12]. This correlation underscores the negative impact of PHS on rehabilitation progress, emphasizing the crucial role early management plays in enhancing sensorimotor integration, facilitating motor function restoration, improving overall outcomes, and reducing readmissions. In our study, we identified various contributing factors, including glenohumeral subluxation, rotator cuff tendinopathies, spasticity, adhesive capsulitis, complex regional pain syndrome, and central pain, in order of prevalence. These etiologies were similarly reported in other studies as major causes of PHS [4,10,20].

The multiplicity of etiologies necessitates a thorough approach to treatment, as distinct underlying mechanisms may require specifically tailored therapeutic interventions. Multiple treatment modalities are employed for this condition with varying levels of evidence, often limited in scope and occasionally presenting conflicting results [7,15].

In our cohort, therapeutic options included conventional physiotherapy, intra-articular corticosteroid injections, botulinum toxin (BT) therapy, suprascapular nerve blocks, and radiofrequency neuromodulation. Among these, corticosteroid injections and electrical stimulation demonstrated significant efficacy in alleviating symptoms and enhancing functional outcomes. The positive outcomes associated with corticosteroid injections are particularly noteworthy, as they provide a minimally invasive option for managing pain and improving mobility. These findings align with previous studies showing corticosteroids to be more effective in pain control than pulsed radiofrequency modulation [21].

Conversely, the efficacy of botulinum toxin therapy in this context remained inconsistent, highlighting the need for further research to clarify its role in PHS management. Variability in treatment responses across patients underscores the necessity of personalized treatment plans that consider individual etiologies and functional statuses.

We propose that incorporating individualized treatments alongside conventional rehabilitation significantly improves outcomes, as evidenced and supported by other studies [22].

Limitations

This study, being retrospective in nature, is inherently subject to certain limitations. The sample size, though considerable, was derived from a single rehabilitation centre, which may limit the generalisability of the findings to broader populations. Furthermore, the reliance on pre-existing medical records posed a challenge, as incomplete or inconsistent data may have affected the comprehensiveness and accuracy of the analysis. Another key limitation is the relatively short follow-up period, which may not fully capture the incidence or progression of PHS, particularly in cases with delayed onset during the recovery process. These factors underscore the need for caution when interpreting and extrapolating the study’s results. Future research should prioritise larger, more diverse cohorts and extended follow-up periods to provide a more robust and nuanced understanding of PHS in post-stroke populations.

## Conclusions

This study provides valuable insights into the incidence, aetiology, and management of PHS in subacute post-stroke patients undergoing inpatient rehabilitation. The findings underscore the multifactorial nature of PHS, with predominant causes such as glenohumeral subluxation, rotator cuff pathology, and adhesive capsulitis, and highlight the effectiveness of tailored interventions like corticosteroid injections and pulsed radiofrequency modulation in alleviating pain and enhancing functional recovery. A multifaceted and individualized therapeutic approach is crucial for improving outcomes and quality of life in PHS patients. 

However, the study’s retrospective design, single-centre scope, and limited follow-up period highlight the need for further research. Future studies should involve larger, multi-centre cohorts with longer follow-ups are essential to deepen understanding of PHS progression and the long-term efficacy of treatment strategies, ultimately enhancing rehabilitation outcomes for post-stroke patients.

## References

[1] Winstein CJ, Stein J, Arena R (2016). Guidelines for adult stroke rehabilitation and recovery: a guideline for healthcare professionals from the American Heart Association/American Stroke Association. Stroke.

[2] Holmes RJ, McManus KJ, Koulouglioti C, Hale B (2020). Risk factors for post-stroke shoulder pain: A systematic review and meta-analysis. J Stroke Cerebrovasc Dis.

[3] Mehta S, Teasell R, Foley N (2013). Painful hemiplegic shoulder. Evidence-Based Review of Stroke Rehabilitation.

[4] Anwer S, Alghadir A (2020). Incidence, prevalence, and risk factors of hemiplegic shoulder pain: a systematic review. Int J Environ Res Public Health.

[5] Poduri KR (1993). Shoulder pain in stroke patients and its effects on rehabilitation. J Stroke Cerbrovasc Dis.

[6] Wanklyn P, Forster A, Young J (1996). Hemiplegic shoulder pain (HSP): natural history and investigation of associated features. Disabil Rehabil.

[7] Vasudevan JM, Browne BJ (2014). Hemiplegic shoulder pain: an approach to diagnosis and management. Phys Med Rehabil Clin N Am.

[8] Hadianfard H, Hadianfard MJ (2008). Predictor factors of hemiplegic shoulder pain in a group of stroke patients. Iran Red Crescent Med J.

[9] Neves AF, Camões Barbosa A (2016). Painful hemiplegic shoulder: from prevention to treatment. Rev Soc Port Med Fís Reabil.

[10] Kalichman L, Ratmansky M (2011). Underlying pathology and associated factors of hemiplegic shoulder pain. Am J Phys Med Rehabil.

[11] Adey-Wakeling Z, Arima H, Crotty M, Leyden J, Kleinig T, Anderson CS, Newbury J (2015). Incidence and associations of hemiplegic shoulder pain poststroke: prospective population-based study. Arch Phys Med Rehabil.

[12] Nadler M, Pauls M, Cluckie G, Moynihan B, Pereira AC (2020). Shoulder pain after recent stroke (SPARS): hemiplegic shoulder pain incidence within 72hours post-stroke and 8-10 week follow-up (NCT 02574000). Physiotherapy.

[13] Li Y, Yang S, Cui L (2022). Prevalence, risk factor and outcome in middle-aged and elderly population affected by hemiplegic shoulder pain: An observational study. Front Neurol.

[14] Murie-Fernández M, Carmona Iragui M, Gnanakumar V, Meyer M, Foley N, Teasell R (2012). Painful hemiplegic shoulder in stroke patients: causes and management [article in Spanish]. Neurologia.

[15] Dyer S, Mordaunt DA, Adey-Wakeling Z (2020). Interventions for post-stroke shoulder pain: an overview of systematic reviews. Int J Gen Med.

[16] Picelli A, Lobba D, Vendramin P (2018). A retrospective case series of ultrasound-guided suprascapular nerve pulsed radiofrequency treatment for hemiplegic shoulder pain in patients with chronic stroke. J Pain Res.

[17] Gamble GE, Barberan E, Laasch HU, Bowsher D, Tyrrell PJ, Jones AK (2002). Poststroke shoulder pain: a prospective study of the association and risk factors in 152 patients from a consecutive cohort of 205 patients presenting with stroke. Eur J Pain.

[18] Zeilig G, Rivel M, Doron D, Defrin R (2016). Does hemiplegic shoulder pain share clinical and sensory characteristics with central neuropathic pain? A comparative study. Eur J Phys Rehabil Med.

[19] Duray M, Baskan E (2020). The effects of hemiplegic shoulder pain on upper extremity motor function and proprioception. NeuroRehabilitation.

[20] Kim YH, Jung SJ, Yang EJ, Paik NJ (2014). Clinical and sonographic risk factors for hemiplegic shoulder pain: A longitudinal observational study. J Rehabil Med.

[21] Kim TH, Chang MC (2021). Comparison of the effectiveness of pulsed radiofrequency of the suprascapular nerve and intra-articular corticosteroid injection for hemiplegic shoulder pain management. J Integr Neurosci.

[22] de Sire A, Moggio L, Demeco A (2022). Efficacy of rehabilitative techniques in reducing hemiplegic shoulder pain in stroke: Systematic review and meta-analysis. Ann Phys Rehabil Med.

